# Changes in Health Perception among Patients with Aortic Diseases in a Severe COVID-19 Area in the West of Germany: A Longitudinal Study between the First and Second Wave of the COVID-19 Pandemic

**DOI:** 10.3390/medicina57090888

**Published:** 2021-08-27

**Authors:** Alan Parys, Sarah Klinge, Alina Dönmez, Christos Rammos, Tienush Rassaf, Rolf Alexander Jánosi, Julia Lortz

**Affiliations:** Department of Cardiology and Vascular Medicine, West-German Heart and Vascular Center Essen, University of Duisburg-Essen, University Clinic of Essen, Hufelandstr. 55, 45147 Essen, Germany; alan.parys@uk-essen.de (A.P.); sarah.klinge@uk-essen.de (S.K.); alina.dirk-doenmez@outlook.com (A.D.); christos.rammos@uk-essen.de (C.R.); tienush.rassaf@uk-essen.de (T.R.); alexander.janosi@uk-essen.de (R.A.J.)

**Keywords:** COVID-19, pandemic, cardiovascular diseases, aortic diseases, health perception

## Abstract

*Background and Objectives:* The rapid spread of the novel coronavirus disease (COVID-19) has become the most challenging global health pandemic since the 1918 flu. In Germany, more than 3.4 million cases are confirmed so far, including 83,000 deaths. Increased fatality rates among patients with pre-existing cardiovascular diseases (CVD) represent this group at particular risk. The aim of this study was to evaluate changes in health perception among patients with aortic diseases during the first (w1) and second wave (w2) of the COVID-19 pandemic in Germany. *Material and Methods:* Patients (*n* = 262) diagnosed with aortic disease participated in telephone interviews during w1 and w2. The perception of COVID-19 as a threat was examined using relevant items of the Brief Illness Perception (BIP) questionnaire. *Results:* The BIP score increased from 9.18 (SD = 7.132) to 14.58 (SD = 6.956) between w1 and w2 (*p* < 0.001). Although this population is at high risk their overall perception of COVID-19 as a threat was low in the beginning, but surged during w2. Main reasons were increased effects on personal life and elevated concerns about the pandemic, but did not include the educational aspect of COVID-19. *Conclusions:* Tailored risk communication strengthens the mental health of people in a public health crisis and ensures the success of governmental guidelines.

## 1. Introduction

The initial outbreak of the novel coronavirus disease (COVID-19) was first reported in Wuhan City in China in December 2019 [1]. A novel betacoronavirus, known as the severe acute respiratory syndrome coronavirus 2 (SARS-CoV-2), was identified as the pathogen for COVID-19 [2]. The virus rapidly spread to other countries worldwide as a global threat and has become the most challenging global health pandemic since the 1918 flu. On 11 March 2020, the WHO finally made the assessment that COVID-19 can be characterized as a pandemic, following 1918 Spanish flu (H1N1), 1957 Asian flu (H2N2), 1968 Hong Kong flu (H3N2), and 2009 Pandemic flu (H1N1), which caused an estimated 50 million, 1.5 million, 1 million, and 300,000 human deaths, respectively [3]. The most common symptoms at onset of COVID-19 illness are fever, cough, and fatigue, while other symptoms include sputum production, headache, and dyspnoea [4]. In February 2021, the World Health Organization confirmed over 30 million cases of COVID-19 for Europe, including nearly 800,000 deaths. In Germany, over 3 million cases were confirmed, with nearly 87,000 deaths [5]. It has been shown that there is an increased case fatality rate among patients with pre-existing comorbid conditions, especially in patients with cardiovascular diseases (CVD) [6]. In general, patients with CVD risk factors may be predisposed to COVID-19, and those who are affected have an increased risk of cardiovascular complications [7]. Furthermore, patients with pre-existing CVD appear to have worse outcomes with COVID-19 [8]. In a case series conducted at the beginning of the epidemic in China, among patients hospitalized for COVID-19, those with comorbidities were more likely to be hospitalized in intensive care units [9]. While no association has been made specifically between COVID-19 and aortic dissection, several reports of myocarditis, ischemic heart disease and vascular injury (i.e., stroke) have been observed [10]. Slowing the spread of COVID-19 is the most important task worldwide. It requires massive action from governments, industry and citizens, where people have to actively change their lives and follow best practices for social distancing and hygiene. At long last the success of regulations depends on citizens’ compliance [11]. According to theories of decisions about health behaviour, people who perceive greater risks are more motivated to implement protective behaviours [12]. On the other hand, quarantine measures during the pandemic are associated with increased risk of experiencing mental health burden, especially for vulnerable groups including people with pre-existing mental or physical illnesses [13,14]. Beginning in mid-March the first nationwide anti-corona measures were implemented in Germany. The second wave started in mid-December. It could be already shown that risk perceptions of public health crises like the COVID-19 pandemic can affect people’s mental health. The aim of the present study was to evaluate changes in health perception among patients with aortic diseases during the first and second wave of the pandemic in a severe COVID-19 area in the west of Germany.

## 2. Materials and Methods

### 2.1. Study Design and Patient Recruitment

**Figure 1:**
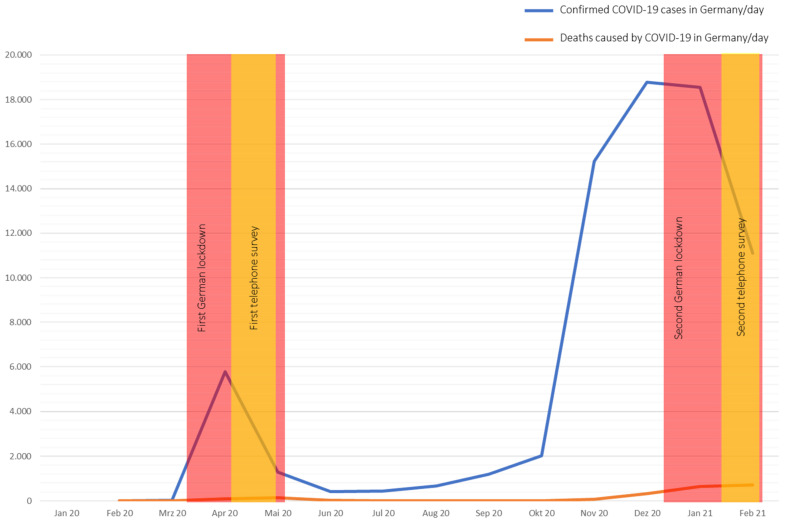
Course of the COVID-19 Pandemic in Germany. (Data are extracted from OurWorldInData.org [5], access date: 28 February 2021).

We conducted a prospective, longitudinal questionnaire-based survey from patients with aortic diseases treated at the cardiac and vascular outpatient clinic of the West-German Heart and Vascular Center Essen of the University Clinic Essen, Germany. Patients were contacted via telephone at two different time points, during the first wave of the coronavirus pandemic (w1: between 6 April 2020 and 29 April 2020) and the second wave (w2: between 11 January 2021 and 29 January 2021) to answer a questionnaire survey (Figure 1).

The inclusion criteria were male or female patients aged 18 or older with aortic disease, that had to be diagnosed at least 6 months prior to the study and who were not infected by COVID-19 at the beginning of the study. Patients were excluded who were unable to complete the questionnaire (e.g., severe dementia or cognitive dysfunction). We also excluded individuals who did not have sufficient knowledge of the German language.

### 2.2. Ethics

The study was conducted in accordance with the Declaration of Helsinki, and the protocol was approved by the local ethics committee at the medical faculty of the University Duisburg-Essen (19-8718-BO, date of approval: 30 January 2020). Patient records were de-identified and analysed anonymously. Written consent was obtained from each patient included in the study.

### 2.3. Measurements

#### 2.3.1. Questionnaire

The used questionnaire included five items, based on the Brief Illness Perception (BIP)-questionnaire that has nine items [15], all of which are answered on a ten-point Likert scale. The shortened version assessed three major categories. This included two items on cognitive illness representations regarding consequences (Item 1) and personal control (Item 3). One item assessed emotional representation in terms of concern (Item 6), and one item assessed illness comprehensibility (Item 7). The questionnaire at w1 surveyed also patients’ personal protection equipment and behaviour, respectively. Answers contained face masks/face shields, gloves, social distancing, staying home and disinfecting hands. The questionnaire at w2 interrogated previous COVID-19 disease, previous quarantine, and reasons for quarantine.

#### 2.3.2. Patient Records

In addition to the questionnaire, basic demographic data and (co)morbidities were taken from the electronic patient records (Medico, Cerner Corporation). Relevant data included age, gender, type of aortic disease, relevant comorbidities focusing on diseases of the arteriosclerotic group and cardiovascular risk factors. Moreover, previous aortic therapies including endovascular treatment and aortic surgery were recorded.

### 2.4. Statistical Analysis

The statistical analysis was performed using SPSS 26 (SPSS Inc., Chicago, IL, USA). Variables were presented as frequencies and percentages or as the means and standard deviations. Correlations were calculated using Pearson’s correlation for metric variables. For correlations between nominal and metric variables, Eta was determined using cross-tabulations and the level of significance was calculated using univariate variance analysis. The two measurement time points were compared using the paired-sample *t*-test. For pairwise group comparisons, the individual dimensions of the BIP score were used and, in addition, a sum score was calculated from these. The two items assessing the dimensions personal control and coherence were inverted to calculate the sum score. Values of *p* < 0.05 were considered statistically significant.

## 3. Results

### 3.1. Descriptive Analysis

#### 3.1.1. Sociodemographic Characteristics

At both time points, 262 patients participated in the study. All of them live in the Ruhr region, which was a severe COVID-19 area in the west of Germany. With a steady increase in the large number of inhabitants it belonged to a high-incidence area during the COVID-19 pandemic constantly. All participants could be included in the analysis. Of these, 182 (69.5%) were male and 80 (30.5%) were female. The participants were between 28 and 92 years old (M = 68.39, SD = 12.96 at w1; M = 68.51, SD = 12.94 at w2). Aortic diseases included mainly aortic aneurysms of the ascending aorta (*n* = 164, 62.6%), but included also aortic aneurysms of the descending aorta (*n* = 37, 14.1%). Patients after diagnosed acute or chronic aortic dissection made up a third (*n* = 41, 15.6%, and *n* = 48, 18.3%, respectively). Patients with diagnosed penetrating aortic ulcer (*n* = 17, 6.5%), and intramural hematoma (*n* = 12, 4.6%) were a minority. A total of 12 patients had diagnosed Marfan disease (4.6%).

Sixty-nine (26.6%) of the participants had endovascular treatment of their previous diagnosed aortic disease, 38 (14.7%) underwent surgery and 152 (58.7%) were treated conventionally. Table 1 shows an overview of the sociodemographic characteristics of the study population.

At baseline, no participants had been neither quarantined nor had COVID-19. At the second survey at w2, 24 participants (9.5%; *n* = 252) had already been quarantined and five (2%; *n* = 252) were infected with COVID-19.

#### 3.1.2. Cardiovascular Risk Factors and Comorbidities

The overall prevalence of classical cardiovascular risk factors was high in the study population. The vast majority had diagnosed arterial hypertension (*n* = 233, 88.9%). High prevalence was also seen for hyperlipidaemia (*n* = 142, 54.2%), and active or a former smoker (*n* = 118, 45%). Adipositas and diabetes mellitus Type II were diagnosed in 62 (23.8%) and 36 patients (13.7%), respectively.

Relevant atherosclerotic comorbidities were coronary artery disease (*n* = 110, 28.1%), including 33 patients with a history of myocardial infarction (12.6%), and peripheral arterial disease (*n* = 30, 11.5%). Twenty-five patients had prior stroke (9.5%). Valvular heart disease was diagnosed in 175 patients (66.8%). Table 2 gives an overview of cardiovascular risk factors and comorbidities.

### 3.2. Correlations with the BIP Sum Score

#### 3.2.1. Sociodemographic Characteristics

At w1, there was no significant correlation between the age and the perception of COVID-19 as a threat (*r* = −0.045, *p* = 0.467). In contrast, gender showed a small positive significant correlation with the perception of COVID-19 as a threat (*r* = 0.155, *p* = 0.012). At w2, neither age (*r* = −0.019, *p* = 0.769) nor gender (*r* = 0.444, *p* = 0.096) showed a significant correlation.

#### 3.2.2. Aortic Disease

At both time points, we assessed whether there was an association between the recorded atherosclerotic diseases and the perception of COVID-19 as a threat. No significant correlations were found for either w1 or w2.

#### 3.2.3. Cardiovascular Risk Factors and Comorbidities

We detected a positive correlation between the BIP sum score and history of myocardial infarction at both time points (r = 0.136, *p* = 0.028, and r = 0.127, *p* = 0.045, respectively). The same was seen in terms of vascular heart disease, whereas at both times a positive correlation was found (r = 0.131, *p* = 0.034, and r = 0.153, *p* = 0.015, respectively).

### 3.3. Comparison between the Two Measure Time Points

#### 3.3.1. BIP Sum Score

At w1, the BIP score had a minimum of 0 and a maximum of 32, with an average score of 9.18 (SD = 7.132). The average score of 14.58 (SD = 6.956) is noticeably higher at w2, but the responses still range from 0 to 32.

To examine whether there is a difference regarding the perception of COVID-19 as a threat at the two measurement time points, we first investigated whether the conditions for calculating a *t*-test were met. There were no outliers, which allowed all participants to be included in the analyses. According to the Kolmogorov–Smirnov test there is a normal distribution of the residuals (*p* = 0.075), but not according to the Shapiro–Wilk test (*p* = 0.016). However, since the *t*-test is considered relatively robust to a violation of a normal distribution (when the sample size is greater than 50), the paired-sample *t*-test was conducted.

According to the paired-sample *t*-test, there was a significant difference regarding the perception of COVID-19 as a threat between w1 and w2 (*M* = −5.393, *SD* = 6.651, 95% CI (−6.218, −4.568), *t* (251) = −12.871, *p* < 0.001, *d* = 0.81). The BIP score was significantly lower at w1 (*M* = 9.18, *SD* = 7.132) than at w2 (*M* = 14.58, *SD* = 6.956), indicating that COVID-19 posed a greater threat to the participants at w2.

#### 3.3.2. BIP Dimensions

Additionally, we examined whether there was a difference at the two time points in terms of the four dimensions surveyed (consequences, personal control, concern, and coherence).

The paired-sample *t*-test showed a significant difference regarding the consequences of the COVID-19 pandemic on one’s life between w1 and w2 (*M* = −2.821, *SD* = 3.049, 95% CI (−3.200, −2.443), *t* (251) = −14.691, *p* < 0.001, *d* = 0.92). The subjectively perceived consequences were lower at w1 (*M* = 1.69, *SD* = 2.774) than at w2 (*M* = 4.52, *SD* = 2.923).

There was also a significant difference in terms of perceived control according to the paired-samples *t*-test (*M* = 0.908, *SD* = 2.492, 95% CI (0.589, 1.218), *t* (249) = 5.760, *p* < 0.001, *d* = 0.36). At w1 (*M* = 7.07, *SD* = 2.346), perceived control of getting sick of COVID-19 was higher than at w2 (*M* = 6.16, *SD* = 2.448) Furthermore, there was a significant difference between w1 and w2 in personal concern about getting COVID-19 because of their aortic disease (*M* = −1.669, *SD* = 3.349, 95% CI (−2.086, −1.253), *t* (250) = −7.898, *p* < 0.001, *d* = 0.50). Concern was lower at w1 (*M* = 3.04, *SD* = 3.661) than at w2 (*M* = 4.71, *SD* = 3.267).

Only in understanding about the disease, there was no significant difference (*M* = −0.032, *SD* = 1.520, 95% CI [−0.220, 0.157], *t* (251) = −0.332, *p* = 0.740) between w1 (*M* = 8.44, *SD* = 1.544) and w2 (*M* = 8.47, *SD* = 1.590).

Yet, the difference of the overall BIP-score between w1 and w2 was significant (*M* = −5.393, *SD* = 6.651, 95% CI (−6.218, −4.568), *t* (251) = 12.871, *p* < 0.001). The overall perception of COVID-19 as a threat was lower at w1 (*M* = 9.18, *SD* = 7.132) than at w2 (*M* = 14.58, *SD* = 6.958). Table 3 provides a detailed overview of the comparison of the BIP dimensions at both waves with the respective means and standard deviations at the time points.

## 4. Discussion

The COVID-19 pandemic has become the most challenging global health thread of the present century. It could be shown that the presence of cardiovascular diseases is associated with a higher risk of mortality when compared to COVID-19 patients without pre-existing cardiovascular diseases. Even coronary disease and other types of cardiovascular diseases, hypertension, and cerebrovascular diseases almost double the risk of mortality [16]. Although the health perception of COVID-19 is a crucial point for the sustainable implementation of personal protective measures and the acceptance of regulatory requirements, little is known about the health perception of COVID-19 among the subpopulation of patients with aortic diseases so far.

During the first wave, the BIP sum score in patients with aortic diseases was within the lower third of the rating scale, indicating a decreased risk perception in terms of COVID-19. When considering the dimensions “control”, “coherence”, “consequence”, and “concern” in detail, all dimensions scored comparably low (“control” and “coherence” after inversion). The inadequate risk perception in a highly endangered cohort seems paradoxical but was already reported previously. A cross-sectional survey showed, that over a third of high-risk patients did not feel at risk of severe COVID-19 infection and therefore adopted incautious attitudes [17]. Our study confirmed the distorted risk perceptions of severe COVID-19 infection in patients with aortic diseases. The underlying reason is highly speculative but might be associated with the general poor understanding of patients’ conditions among aortic diseases, as previously shown for patients with abdominal aortic aneurysms [18].

During the second wave, we found a significant rise in the BIP sum score indicating an increased perception of COVID-19 as a threat among patients with aortic diseases. The loss of personal control, the associated consequences and perceived restrictions in daily life, and the increased concerns scored relevant higher in patients with aortic diseases compared to w1. The continuous presence of COVID-19 in the media and the resulting tightening measures by the government in Germany, including a lockdown that lasted 4 weeks, might be potential trigger for the rise. The measures taken by the government, such as lockdown and quarantine, may have heavily interfered with the individual choices of the study population, resulting in a variation of their daily behaviours and habits. Lockdown, quarantine, and social distancing resulting from the continuous escalation of the pandemic cause people to worry, feel anxious, and perceive themselves at risk of being taken ill with COVID-19. Undoubtedly, the COVID-19 pandemic has a significant impact on psychosocial and physical health of individuals in general [19] and the elevated risk perception of COVID-19 is negatively related to psychological well-being [20]. Even the general public shows increased symptoms of depression, anxiety, and stress related to COVID-19, as a result of psychosocial stressors such as life disruption or fear of illness. The COVID-19 pandemic has already affected mental health, and some of these effects might persist. Especially during pandemics new therapeutic methods like telehealth with remote video or phone conferencing, online blended or coached therapies, and self-help therapies should be established more intense to prevent people with unfavourable comorbid conditions from getting diseased [21].

Noticeably despite the increase of the BIP sum score, the reported knowledge about the COVID-19 virus remained widely stable. In case of the newly emerged mutants of COVID-19 [22,23] information was widely spread through (social) media ensuring a high level of public knowledge [24,25], but increased also the perception of COVID-19 as a threat through a certain unpredictability of the further dynamics of the pandemic.

Knowledge and attitudes of patients with chronic conditions are key features in risk perception of COVID-19 [26,27], and despite disagreements about the risks, people perceiving greater risks were more likely to implement protective behaviours [13]. The current gaps in awareness of COVID-19 among patients with aortic diseases are essential to address in the context and their attitudes to prevent infection.

Decision makers, who are usually prone to act according to the effective risk, should consider the public perception of health risk. Health care providers have the special task of personalized risk communication in endangered subpopulations, since this is crucial for an effective emergency and risk management, and ensures the acceptance of personal protective measures and the implementation of governmental requirements.

Due to the rapid dynamics of the pandemic, regulatory requirements for protective measures tightened during the study. Thus, keeping distance and wearing masks in public became mandatory in Germany and violations were punished as administrative offenses. As a result, the item on personal protective measures were removed for the second survey. Despite the relatively large sample size for aortic diseases in general, some aortic disease patterns are only sparsely represented (e.g., penetrating aortic ulcer) and do not allow any conclusions to be drawn about the special subgroup.

## 5. Conclusions

Patients with aortic diseases are highly endangered referred to COVID-19 and their overall perception of COVID-19 as a threat was low during the first wave but increased during the second. The main reasons were the increased effects on personal life and elevated concerns about the COVID-19 pandemic, but concerns did not include the educational aspect of COVID-19.

In conclusion, we found a distorted risk perception in terms of COVID-19 among patients with aortic disease, especially at the beginning of the pandemic. Targeted and personalized risk communication may increase the use of personal protective equipment and the acceptance of governmental requirements.

## Figures and Tables

**Table 1:** Sociodemographic characteristics.

	Study Population
	(*n* = 262)
Male gender, *n* (%)	182 (69.5)
Age (years), mean ± SD	68.4 ± 12.9
Diagnosed aortic disease	
Acute aortic dissection, *n* (%)	41 (15.6)
Chronic aortic dissection, *n* (%)	48 (18.3)
Intramural hematoma, *n* (%)	12 (4.6)
Penetrating aortic ulcer, *n* (%)	17 (6.5)
Aortic aneurysm	
Ascending aorta, *n* (%)	164 (62.6)
Descending aorta, *n* (%)	37 (14.1)
Previous aortic treatment	
Open surgery, *n* (%)	38 (14.5)
Endovascular treatment, *n* (%)	69 (26.3)
Conventional treatment, *n* (%)	155 (59.1)
Re-Intervention, *n* (%)	39 (14.9)
ICU stay referred to aortic disease, *n* (%)	95 (36.3)

ICU, intensive care unit; SD, standard deviation.

**Table 2:** Cardiovascular risk factors and comorbidities.

	Study Population
	(*n* = 262)
Cardiovascular risk factors	
Arterial hypertension, *n* (%)	233 (88.9)
Hyperlipidaemia, *n* (%)	142 (54.2)
Active or former smoking, *n* (%)	118 (45)
Obesity (BMI > 30 kg/m^2^), *n* (%)	62 (23.8)
Diabetes mellitus type II, *n* (%)	36 (13.7)
Relevant comorbidities	
CAD, *n* (%)	110 (28.1)
Previous coronary intervention, *n* (%)	59 (22.5)
Previous CABG, *n* (%)	27 (10.3)
Previous myocardial infarction, *n* (%)	33 (12.6)
PAD, *n* (%)	30 (11.5)
Previous stroke, *n* (%)	25 (9.5)
Valvular heart disease, *n* (%)	175 (66.8)
Bicuspid aortic valve, *n* (%)	17 (6.5)
Previous valve replacement, *n* (%)	41 (15.6)
COPD, *n* (%)	32 (12.2)
Renal insufficiency, *n* (%)	49 (18.7)

BMI, body mass index; CABG, coronary arterial bypass grafting; CAD, coronary arterial disease; COPD, chronic obstructive pulmonary disease; PAD, peripheral arterial disease.

**Table 3:** Comparison of the BIP sum at the first (w1) and second (w2) wave.

	First Wave		Second Wave						
	04/06–04/29/2020		01/11–01/29/2021		*T* (251)	*p*	CI 95%		Cohen’s *d*
	*M*	*SD*	*M*	*SD*			Lower	Upper	
Overall BIP-score	9.18	7132	14.58	6956	12,871	<0.001	−6.218	−4.568	0.81
*BIP Dimensions*									
Consequences	1.69	2774	4.52	2923	14,691	<0.001	−3.200	−2.443	0.92
Personal Control	7.07	2346	6.16	2448	5.760 *	<0.001	0.589	1,218	0.36
Concern	3.04	3661	4.71	3267	−7.898 *	<0.001	−2.086	−1.253	0.50
Coherence	8.44	1544	8.47	1590	−0.332	0.740	−0.220	0.157	−0.02

BIP, brief illness perception questionnaire; M, median; SD, standard deviation. * *t* (250).

## Data Availability

The data presented in this study are available on request from the corresponding author. The data are not publicly available due to privacy and ethics.

## References

[1] Wang C., Horby P.W., Hayden F.G., Gao G.F. (2020). A novel coronavirus outbreak of global health concern. Lancet.

[2] Drosten C., Gunther S., Preiser W., van der Werf S., Brodt H.R., Becker S., Rabenau H., Panning M., Kolesnikova L., Fouchier R.A. (2003). Identification of a novel coronavirus in patients with severe acute respiratory syndrome. N. Engl. J. Med..

[3] Liu Y.C., Kuo R.L., Shih S.R. (2020). COVID-19: The first documented coronavirus pandemic in history. Biomed. J..

[4] Rothan H.A., Byrareddy S.N. (2020). The epidemiology and pathogenesis of coronavirus disease (COVID-19) outbreak. J. Autoimmun..

[5] Roser M.R.M., Ortiz-Ospina E., Hasell J. (2020). Coronavirus Pandemic (COVID-19). Our World Data.

[6] Wu Z., McGoogan J.M. (2020). Characteristics of and Important Lessons from the Coronavirus Disease 2019 (COVID-19) Outbreak in China: Summary of a Report of 72314 Cases from the Chinese Center for Disease Control and Prevention. JAMA.

[7] Ashur C., Norton E., Farhat L., Conlon A., Willer C., Froehlich J.B., Pinsky D.J., Kim K.M., Fukuhara S., Deeb M.G. (2020). Higher admission rates and in-hospital mortality for acute type A aortic dissection during Influenza season: A single center experience. Sci. Rep..

[8] Driggin E., Madhavan M.V., Bikdeli B., Chuich T., Laracy J., Biondi-Zoccai G., Brown T.S., Der Nigoghossian C., Zidar D.A., Haythe J. (2020). Cardiovascular Considerations for Patients, Health Care Workers, and Health Systems during the COVID-19 Pandemic. J. Am. Coll. Cardiol..

[9] Cao J., Hu X., Cheng W., Yu L., Tu W.J., Liu Q. (2020). Clinical features and short-term outcomes of 18 patients with corona virus disease 2019 in intensive care unit. Intensive Care Med..

[10] Cao J., Tu W.J., Cheng W., Yu L., Liu Y.K., Hu X., Liu Q. (2020). Clinical Features and Short-term Outcomes of 102 Patients with Coronavirus Disease 2019 in Wuhan, China. Clin. Infect. Dis..

[11] Harper C.A., Satchell L.P., Fido D., Latzman R.D. (2020). Functional Fear Predicts Public Health Compliance in the COVID-19 Pandemic. Int. J. Ment. Health Addict..

[12] Janz N.K., Becker M.H. (1984). The Health Belief Model: A decade later. Health Educ. Q..

[13] Bruine de Bruin W., Bennett D. (2020). Relationships Between Initial COVID-19 Risk Perceptions and Protective Health Behaviors: A National Survey. Am. J. Prev. Med..

[14] Wang Y., Shi L., Que J., Lu Q., Liu L., Lu Z., Xu Y., Liu J., Sun Y., Meng S. (2021). The impact of quarantine on mental health status among general population in China during the COVID-19 pandemic. Mol. Psychiatry.

[15] Broadbent E., Petrie K.J., Main J., Weinman J. (2006). The brief illness perception questionnaire. J. Psychosom. Res..

[16] Moula A.I., Micali L.R., Matteucci F., Lucà F., Rao C.M., Parise O., Parise G., Gulizia M.M., Gelsomino S. (2020). Quantification of Death Risk in Relation to Sex, Pre-Existing Cardiovascular Diseases and Risk Factors in COVID-19 Patients: Let’s Take Stock and See Where We Are. J. Clin. Med..

[17] Tran V.T., Ravaud P. (2020). COVID-19-related perceptions, context and attitudes of adults with chronic conditions: Results from a cross-sectional survey nested in the ComPaRe e-cohort. PLoS ONE.

[18] Suckow B., Schanzer A.S., Hoel A.W., Wyers M., Marone L.K., Veeraswamy R.K., Nolan B.W. (2016). A national survey of disease-specific knowledge in patients with an abdominal aortic aneurysm. J. Vasc. Surg..

[19] Yildirim M., Guler A. (2020). Factor analysis of the COVID-19 Perceived Risk Scale: A preliminary study. Death Stud..

[20] Krok D., Zarzycka B. (2020). Risk Perception of COVID-19, Meaning-Based Resources and Psychological Well-Being amongst Healthcare Personnel: The Mediating Role of Coping. J. Clin. Med..

[21] Moreno C., Wykes T., Galderisi S., Nordentoft M., Crossley N., Jones N., Cannon M., Correll C.U., Byrne L., Carr S. (2020). How mental health care should change as a consequence of the COVID-19 pandemic. Lancet Psychiatry.

[22] Pachetti M., Marini B., Benedetti F., Giudici F., Mauro E., Storici P., Masciovecchio C., Angeletti S., Ciccozzi M., Gallo R.C. (2020). Emerging SARS-CoV-2 mutation hot spots include a novel RNA-dependent-RNA polymerase variant. J. Transl. Med..

[23] Becerra-Flores M., Cardozo T. (2020). SARS-CoV-2 viral spike G614 mutation exhibits higher case fatality rate. Int. J. Clin. Pract..

[24] Ali K.F., Whitebridge S., Jamal M.H., Alsafy M., Atkin S.L. (2020). Perceptions, Knowledge, and Behaviors Related to COVID-19 among Social Media Users: Cross-Sectional Study. J. Med. Internet Res..

[25] Li X., Liu Q. (2020). Social Media Use, eHealth Literacy, Disease Knowledge, and Preventive Behaviors in the COVID-19 Pandemic: Cross-Sectional Study on Chinese Netizens. J. Med. Internet Res..

[26] Ding Y., Du X., Li Q., Zhang M., Zhang Q., Tan X., Liu Q. (2020). Risk perception of coronavirus disease 2019 (COVID-19) and its related factors among college students in China during quarantine. PLoS ONE.

[27] Lee Y.C., Wu W.L., Lee C.K. (2021). How COVID-19 Triggers Our Herding Behavior? Risk Perception, State Anxiety, and Trust. Front. Public Health.

